# Epidemiological survey of echinococcosis in Tibet Autonomous Region of China

**DOI:** 10.1186/s40249-019-0537-5

**Published:** 2019-04-28

**Authors:** Bin Li, Gongsang Quzhen, Chui-Zhao Xue, Shuai Han, Wei-Qi Chen, Xin-Liu Yan, Zhong-Jie Li, M. Linda Quick, Yong Huang, Ning Xiao, Ying Wang, Li-Ying Wang, Gesang Zuoga, Bianba, Gangzhu, Bing-Cheng Ma, Gasong, Xiao-Gang Wei, Niji, Can-Jun Zheng, Wei-Ping Wu, Xiao-Nong Zhou

**Affiliations:** 1 Tibet Autonomous Region Center for Diseases Control and Prevention, Lhasa, 850 000 Tibet Autonomous Region China; 2 National Institute of Parasitic Diseases, Chinese Center for Disease Control and Prevention, Chinese Center for Tropical Diseases Research, WHO Collaborating Centre for Tropical Diseases, National Center for International Research on Tropical Diseases, Ministry of Science and Technology, Key Laboratory of Parasite and Vector Biology, MOH, Huangpu District, Shanghai, 200 025 China; 3 Henan Center for Diseases Control and Prevention, Zhengzhou, Shanghai, 450 000 Henan China; 4 Yunnan Institute of Diseases Control and Prevention, Kunming, 650 000 Yunnan China; 5 Chinese Center for Diseases Control and Prevention, Changping, Beijing, 102 200 China; 6 Center for Diseases Control and Prevention, Atlanta, GA 30 328 USA; 7 Shandong Institute of Parasitic Diseases, Jining, 272 033 Shandong China; 8 Lhasa Center for Diseases Control and Prevention, Lhasa, 850 000 Tibet Autonomous Region China; 9 Shigatse Center for Diseases Control and Prevention, Sangzhuzi District, 857 000 Tibet Autonomous Region China; 10 Shannan Center for Diseases Control and Prevention, Shannan, 856 000 Tibet Autonomous Region China; 11 Linzhi Center for Diseases Control and Prevention, Linzhi, 860 000 Tibet Autonomous Region China; 12 Changdu Center for Diseases Control and Prevention, Changdu, 854 000 Tibet Autonomous Region China; 13 Naqu Center for Diseases Control and Prevention, Naqu, 852 000 Tibet Autonomous Region China; 14 Ali Center for Diseases Control and Prevention, Ali, 859 000 Tibet Autonomous Region China; 15 Beijing, China

**Keywords:** Tibet, Echinococcosis, Prevalence, Ultrasonography

## Abstract

**Background:**

The echinococcosis is prevalent in 10 provinces /autonomous region in western and northern China. Epidemiological survey of echinococcosis in China in 2012 showed the average prevalence of four counties in Tibet Autonomous Region (TAR) is 4.23%, much higher than the average prevalence in China (0.24%). It is important to understand the transmission risks and the prevalence of echinococcosis in human and animals in TAR.

**Methods:**

A stratified and proportionate sampling method was used to select samples in TAR. The selected residents were examined by B-ultrasonography diagnostic, and the faeces of dogs were tested for the canine coproantigen against *Echinococcus* spp*.* using enzyme-linked immunosorbent assay. The internal organs of slaughtered domestic animals were examined by visual examination and palpation. The awareness of the prevention and control of echinococcosis among of residents and students was investigated using questionnaire. All data were inputted using double entry in the Epi Info database, with error correction by double-entry comparison, the statistical analysis of all data was processed using SPSS 21.0, and the map was mapped using ArcGIS 10.1, the data was tested by Chi-square test and Cochran-Armitage trend test.

**Results:**

A total of 80 384 people, 7564 faeces of dogs, and 2103 internal organs of slaughtered domestic animals were examined. The prevalence of echinococcosis in humans in TAR was 1.66%, the positive rate in females (1.92%) was significantly higher than that in males (1.41%), (*χ*^2^ = 30.31, *P* < 0.01), the positive rate of echinococcosis was positively associated with age (*χ*^2^_trend_ = 423.95, *P* < 0.01), and the occupational populations with high positive rates of echinococcosis were herdsmen (3.66%) and monks (3.48%). The average positive rate of *Echinococcus* coproantigen in TAR was 7.30%. The positive rate of echinococcosis in livestock for the whole region was 11.84%. The average awareness rate of echinococcosis across the region was 33.39%.

**Conclusions:**

A high prevalence of echinococcosis is found across the TAR, representing a very serious concern to human health. Efforts should be made to develop an action plan for echinococcosis prevention and control as soon as possible, so as to control the endemic of echinococcosis and reduce the medical burden on the population.

**Electronic supplementary material:**

The online version of this article (10.1186/s40249-019-0537-5) contains supplementary material, which is available to authorized users.

## Multilingual abstracts

Please see Additional file 1 for translations of the abstract into the five official working languages of the United Nations.

## Background

Echinococcosis is a zoonotic parasitic disease caused by *Echinococcus* spp*.* as it parasitizes humans or animals, and is globally distribution [1, 2]. Echinococcosis is one of the 17 neglected tropical diseases recognized by the World Health Organization (WHO) and one of the neglected-zoonoses that the WHO prioritizes in their support of prevention and control. According to WHO, the global disease burden caused by echinococcosis was approximately 871 000 disability-adjusted life-years (DALYs) in 2010 [3]. In addition, cystic echinococcosis caused an annual economic loss of approximately USD 2 billion in the livestock farming industry [4]. Echinococcosis in humans presents predominantly as the cystic or alveolar types. Cystic echinococcosis (CE) caused by *Echinococcus granulosus* is mainly transmitted between livestock (intermediate hosts), such as yak and sheep, and dogs (definitive host). The dogs are infected after eating the viscera of the diseased livestock and excrete the echinococcus eggs in their faeces, which may cause disease in livestock such as yak and sheep after ingestion of the eggs. Alveolar echinococcosis (AE) caused by *Echinococcus multilocular* is mainly circulated in rodents, foxes, and wolves. People can be infected by AE while they eating eggs of *Echinococcus granulosus*. CE is widely distributed, primarily in South America, southern and eastern Africa, Australia, the Mediterranean Basin, central Asia, and China. In some of the areas with the greatest prevalence, such as Peru, Argentina, eastern Africa, and central Asia, the prevalence in localized areas is as high as 5–10% [5]. AE prevalence is mainly constrained to the northern hemisphere. The areas with the high incidence are mainly Alaska, northern and central Europe, Central Asia, Siberia, China and Japan [6, 7].

The epidemic of hydatidosis is serious in western and northern China [8–10]. The Tibet Autonomous Region (TAR) is located in the south-western part of the Qinghai-Tibet Plateau, which has an average elevation of over 4000 m. The region has seven prefectures and a total of 74 counties under its jurisdiction, with a grassland area of 650 000 ha, accounting for 56.72% of the total land area in the region. Farming and grazing yaks, sheep and other livestock are the primary livelihood. It is a heavy epidemic areas with echinococcosis in TAR. B-ultrasonography for self-selected study subjects in Dingqing County and Dangxiong County in Tibet in 2007 showed that the prevalence of echinococcosis were 4.7 and 9.9% [11], respectively. Although rapid tests for echinococcosis are under development, the gold standard for diagnosis is still reliant on ultrasonography. However, due to the lack of technology, poor diagnostic availability, vast territory, and inconvenient transportation in Tibet, there are only sporadic cases reported by the local medical institutions, and no large-scale survey has been conducted until now. These results may have been artificially inflated due to selected bias. In 2012, a stratified random sampling method was used to screen for echinococcosis in Baqin, Cuoqin, Yadong and Nyingchi counties in Tibet, and the results showed that the average prevalence in the four counties was 4.23%.

## Materials and methods

### Survey on echinococcosis prevalence in humans

#### Sampling

The Tibet region has a total of 74 counties in seven prefectures that include 692 townships and 5260 villages. The region has a total population of 3 million. After consulting senior statistician, the sample number is about 2–3% of the total population in TAR. In accordance with the main mode of production of the local residents, all villages were classified into Pastoral area (animal husbandry only), semi-Pastoral and semi-Farm area (animal husbandry and farming both), Farm area (farming only), urban areas (live in urban). A stratified and proportionate sampling method was adopted, and the survey was conducted from August to November 2016. A total of 380 villages were selected in Tibet based on the population of the county: we selected 16 villages, eight villages, four villages and two villages respectively in counties with a population of more than 100 000 50 000–100 000 10 000–50 000, and below 10 000. In a county, the villages were randomly selected, and the number of different modes villages (or sub districts) were determined based on the proportion of the four types of village modes. At least 200 people in the selected village were examined randomly, with the total participants in the survey accounting for 2.5% of the total population of Tibet.

#### Population screening and case diagnosis

The survey team, with help from the local government, set up diagnostic ultrasound sites in the clinics of the local villages to organize the process of surveying the residents. For residents living in more rural areas, surveys were conducted at their homes. As much as possible, all residents in the surveyed villages were included in the survey. More than 200 people in each surveyed village received an abdominal B-ultrasonography examination. Cases were diagnosed and classified according to the “Diagnostic criteria for echinococcosis” of China (WS 257–2006) (the standard is in line with that of WHO), and the imaging data were saved. Serum samples were collected from the cases suspected by B-ultrasonography, and *Echinococcus* antibodies were tested for by enzyme-linked immunosorbent assay (ELISA, Zhuhai Hai Tai biopharmaceutical Co., Ltd. Zhuhai, China) as an auxiliary diagnostic test, specificity and sensitivity of this ELISA test are known to be lower than needed to use as a single diagnostic test.

### Survey of infections among dogs

In the selected 380 villages (or subdistrict), 20 households with dogs were randomly selected guided by the village head in each village. Only one fresh dog faecal sample was collected randomly in the selected household. The collected faecal samples were stored in a freezer under − 70 °C for at least 72 h to inactivate potential eggs. All dog feces were tested for Echinococcus coproantigens by sandwich ELISA, and the test kit had been tested with sensitivity and specificity over 80% (43/45, 45/45, respectively).

### Survey of echinococcosis among livestock

In the selected 380 villages, either yaks, sheep or pigs were tested. During this slaughtering season (October–November), either five yaks or 10 pigs/sheep slaughtered by villagers were selected from each village to be tested. A clinical exam using visual inspection and palpation of the livers, lungs and other organs of the animals were carry out by veterinarians to determine likelihood of echinococcosis. Animals which is positive for echinococcosis will commonly have cysits on the surface of their liver and lung lobes. Parenchyma were palpated and anatomised to check cuticle or protoscolex under microscope.

### Survey of knowledge regarding the prevention and control of echinococcosis

In the selected 380 villages, at least 20 local residents in each village were surveyed randomly using a short questionnaire during home visits. Simultaneously, we surveyed 50 students randomly in the grades of 4, 5 and 6 in primary school with the questionnaire in each county, the questionnaire survey evaluated knowledge regarding the prevention and control of echinococcosis.

### Quality control

#### Training

Before the field survey, all investigators involved in the survey were trained in B-ultrasonography diagnosis, administering questionnaires, conducting laboratory tests and the performing diagnostic methods to recognize livestock diseases of echinococcosis.

#### Determining the surveyed subjects

For the surveyed villages, if the population of the administrative village was large, a smaller village was selected as the surveyed area. If the population of the administrative village was too small and could not meet the requirement of 200 people in the survey, the people from the adjacent village were added to the survey subjects.

#### Case review

It is determined the diagnosis based on the imaging and result of serological test to confirm a case of echinococcosis by the expert team that is formed by the clinical and imaging experts of echinococcosis in China. A confirmed case was a person who had a typical image of echinococcosis or who had an atypical image but with positive result of serological test.

#### Data processing and analysis

The positive rate of echinococcosis in human = the number of diagnosed patients / the number of people examined × 100%.

The positive rate of echinococcosis in livestock = the number of diseased animals / the number of livestock checked × 100%.

The positive rate of the *Echinococcus* coproantigen test = the number of the dogs with positive results / the number of dogs in the test × 100%.

The passed rate of the knowledge and control of echinococcosis = the number of people who passed the test / the number of people included in the test × 100%.

Individuals passed the test if, from the five questions about the prevention and treatment of echinococcosis, three responses were correct.

Prevalence of the population was calculated according to the following equation:

$$ p=\sum \limits_{j= 1}\frac{njwj}{Nj}=\sum \limits_{j=1} pjwj $$

where *p* is the prevalence of the population in the surveyed area, *n* is the number of patients detected in this layer, *N* is the number of surveyed people included in this layer, *j* is the rank of stratification, *w*_*j*_ is the weight of the *j*^th^ stratification (the proportion of the population in the *j*^th^ layer to the total population of the region), and *p*_*j*_ is the positive rate for the *j*^th^ layer.

All data were inputted using double entry in the Epi Info database, with error correction by double-entry comparison. The statistical analysis of all data was processed using SPSS 21.0 (IBM, New York, USA), and the map was mapped using ArcGIS 10.1(ESRI, RedLands, USA), the data was tested by Chi-square test and Cochran-Armitage trend test, and the significant level is *P* < 0.05.

## Results

### B-ultra0073onography screening of echinococcosis in human

We conducted B-ultrasonography examination for 80 384 people in 380 villages of 74 counties in Tibet, including 34 297 males and 46 087 females; the average age was 36 years (1–99). For these individuals surveyed, 99% were ethnically Tibetan. Echinococcosis was diagnosed in 1371 patients, and the overall prevalence in the population of the 74 counties in Tibet was 1.66%. Among them, the case numbers of CE, AE and unidentified type were 1202, 153, and 16, respectively. CE cases accounting for 87.67%, and AE cases accounting for 11.16%. Among six types of CE cases, the cases of active CL and CE1-CE2, transitional CE3 and inactive CE4-CE5 accounted for 36.2, 13.7 and 50.1%, respectively. (Table 1, Table 2)

**Table 1:** Number of villages in counties

Prefecture	County	No. of villages selected
Ali	Cuoqin	4
	Gaer	4
	Gaize	4
	Geji	4
	Pulan	2
	Ritu	2
	Zhada	2
	Subtotal	22
Changdu	Basu	4
	Bianba	4
	Chaya	4
	Dingqing	8
	Gongjue	4
	Jiangda	8
	Karuo	16
	Leiwuqi	4
	Luolong	4
	Mangkang	8
	Zuogong	4
	Subtotal	68
Lhasa	Chengguan	16
	Dazi	4
	Dangxiong	4
	Duilongdeqing	8
	Linzhou	8
	Mozhugongka	4
	Nimu	4
	Qushui	4
	Subtotal	52
Linzhi	Bomi	4
	Chayu	4
	Gongbujiangda	4
	Langxian	4
	Linzhi	4
	Milin	4
	Motuo	4
	Subtotal	28
Naqu	Anduo	4
	Baqing	4
	Bange	4
	Biru	8
	Jiali	4
	Naqu	16
	Nima	4
	Nierong	4
	Shenzha	4
	Shuanghu	2
	Suoxian	4
	Subtotal	58
Shigatse	Angren	8
	Bailang	4
	Dingjie	4
	Dingri	8
	Gangba	4
	Jilong	4
	Jiangzi	8
	Kangma	4
	Lazi	4
	Nanmulin	9
	Nielamu	4
	Renbu	4
	Saga	4
	Sajia	4
	Sangzhuzi	16
	Xietongmen	4
	Yadong	3
	Zhongba	4
	Subtotal	100
Shannan	Cuomei	4
	Cuona	4
	Gongga	4
	Jiacha	4
	Langkazi	4
	Longzi	4
	Luozha	4
	Naidong	8
	Qiongjie	4
	Qusong	4
	Sangri	4
	Zhanang	4
	Subtotal	52
Total		380

**Table 2:** Prevalence of AE and CE echinococcosis across populations in Tibet Autonomous Region, 2016

Prefecture	Total population	Surveyed population	CE Prevalence/% (95% *CI*)	AE Prevalence/% (95% *CI*)	CE & AE Prevalence/% (95% *CI*)
Lhasa	559 423	10 917	1.05 (0.86–1.24)	0.22 (0.13–0.31)	1.27 (1.06–1.48)
Changdu	657 505	14 289	1.22 (1.04–1.40)	0.28 (0.19–0.37)	1.50 (1.30–1.70)
Shannan	328 990	11 184	1.31 (1.10–1.52)	0.04 (0.00–0.08)	1.35 (1.14–1.56)
Shigatse	703 292	21 497	1.00 (0.87–1.13)	0.10 (0.06–0.14)	1.11 (0.97–1.25)
Naque	462 381	11 897	2.98 (2.67–3.29)	0.30 (0.20–0.40)	3.37 (3.05–3.69)
Ali	95 465	4740	1.95 (1.56–2.34)	0.27 (0.12–0.42)	2.31 (1.88–2.74)
Linzhi	195 109	5860	1.34 (1.05–1.63)	0.20 (0.09–0.31)	1.55 (1.23–1.87)
Total	3 002 165	80 384	1.45 (1.37–1.53)	0.20 (0.17–0.23)	1.66 (1.57–1.75)

*CE* Cystic echinococcosis;

*AE* Alveolar echinococcosis

*CI* Confidence interval

### Geographical distribution

**Fig. 1:**
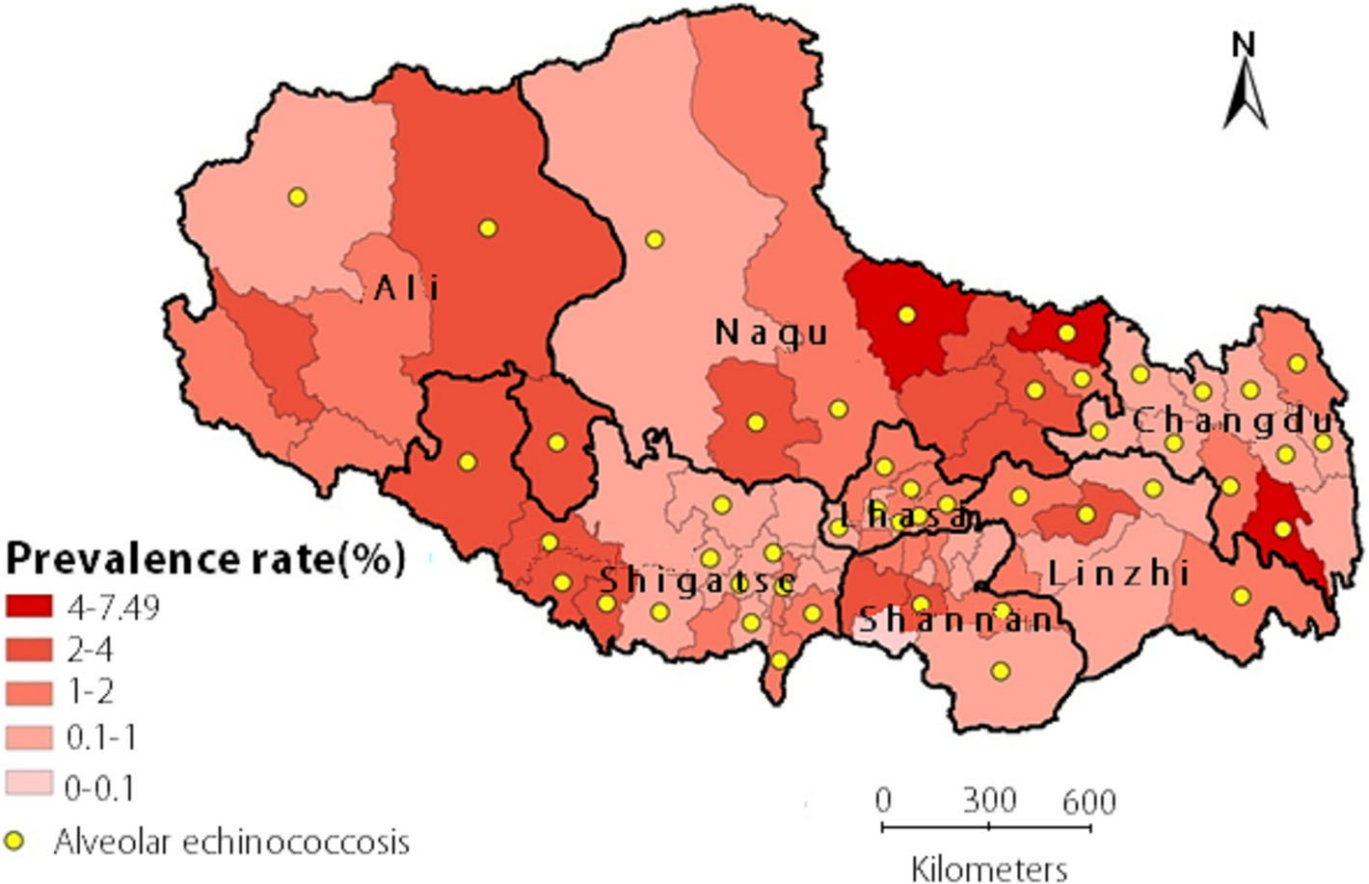
Prevalence distribution by county of AE and CE echinococcosis in Tibet Autonomous Region, 2016. Note: Yellow dot represents counties where AE found

Echinococcosis was detected in all 7 prefectures across the region, with the highest prevalence in Naqu Prefecture (3.37%) and the lowest prevalence in Shigatse Prefecture (1.11%). At the county level, the highest prevalence was found in Zuogong County of Changdu Prefecture (7.82%), while the lowest prevalence was 0.2%. The number of the counties with prevalence 0–0.1% 0.1–1%, 1–2, and 2% or over were 0, 24, 30, and 20, respectively. CE was found in all 74 counties, whereas AE was detected in 47 counties (64%) (Fig. 1, Table 2).

Distribution by gender and age.

**Fig. 2:**
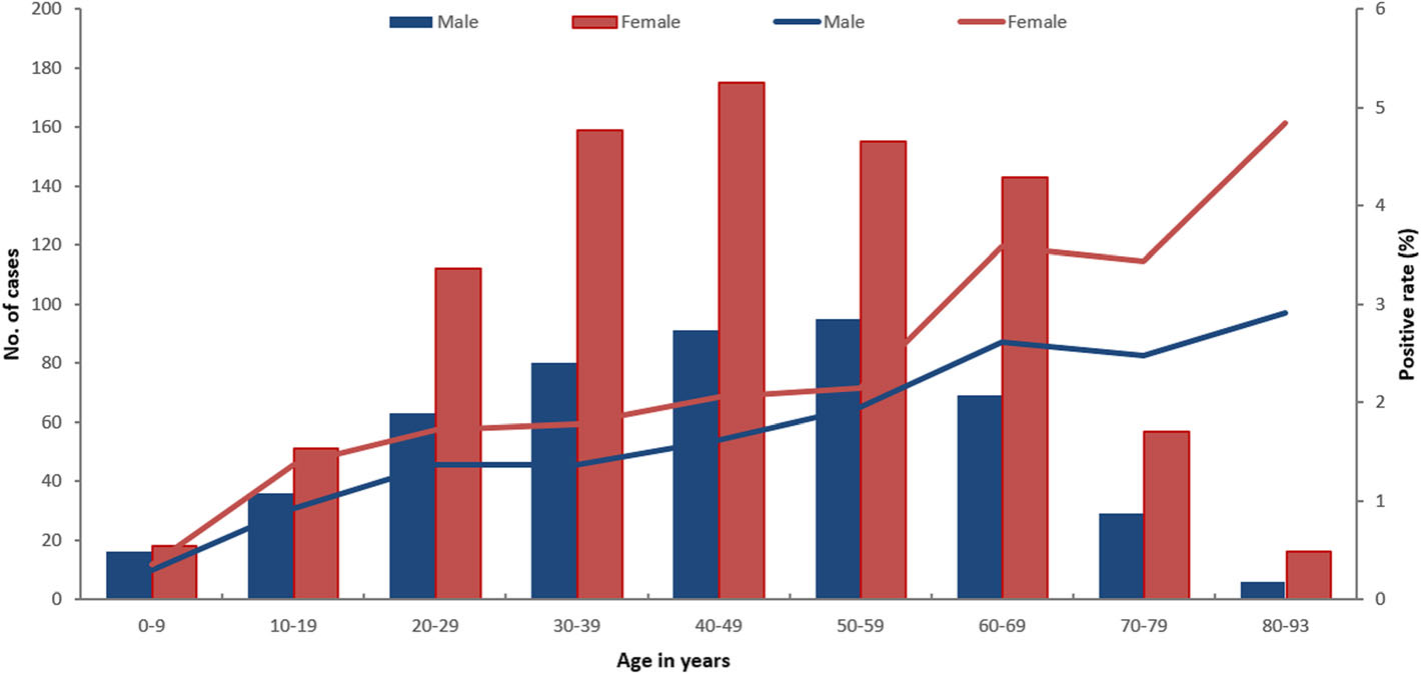
Distribution of AE and CE echinococcosis across different gender and age groups in Tibet Autonomous Region, 2016

A total of 46 087 females in Tibet were examined, and echinococcosis was detected in 886, for a positive rate of 1.92%. A total of 34 297 males in Tibet were examined, and echinococcosis was detected in 485, for a positive rate of 1.41%. The proportion of cases aged 30–59 in both male and female were 55%. The highest proportion of male and female cases were 50–59 and 40–49 age group, respectively (Fig. 2). Statistical testing showed that the positive rate in females was significantly higher than that in males (*χ*^2^ = 30.31, *P* < 0.01). For the echinococcosis patients, the youngest age was 2 years old, and the oldest was 93 years old with a median age of 46 years. The positive rate of echinococcosis in both males and females increased with age (*χ*^2^_trend_ = 423.95, *P* < 0.01).

#### Distribution by occupation, education level and type of production modes

Among the occupational groups, herdsmen (3.66%) and monks (3.48%) showed a higher positive rates. The positive rates of echinococcosis across populations with different education levels were significantly different (*χ*^2^ = 103.19, *P* < 0.01), with the positive rate of echinococcosis of the illiterate group being the highest (2.10%). The positive rates of echinococcosis across the different types of area among those surveyed were significantly different (*χ*^2^ = 168.134, *P* < 0.01), with the pastoral areas showing the highest rates (2.63%) (Table 3).

**Table 3:** Positive rates of AE and CE echinococcosis among different occupations, education levels, and type of area in Tibet Autonomous Region, 2016

	Surveyed population	No. of cases	Constituent ratio/%	Positive rate/% (95% *CI*)
Occupation				
Herdsman	18 759	686	50.04	3.66 (3.39–3.93)
Monks	345	12	0.88	3.48 (1.55–5.41)
Farmer/herdsman	10 078	175	12.76	1.74 (1.48–2.00)
Houseworker	879	12	0.88	1.37 (0.60–2.14)
Farmer	31 369	364	26.55	1.16 (1.04–1.28)
Student	9467	79	5.76	0.83 (0.65–1.01)
Public officer	2104	21	1.53	1.00 (0.57–1.43)
Others	7383	22	1.60	0.30 (0.18–0.42)
Educational level				
Illiterate	46 112	968	70.61	2.10 (1.97–2.23)
Elementary school	26 331	303	22.10	1.15 (1.02–1.28)
Junior high school	5383	69	5.03	1.28 (0.98–1.58)
High school	970	17	1.24	1.75 (0.92–2.58)
College and above	1588	14	1.02	0.88 (0.42–1.34)
Type of area				
Pastoral area	25 098	660	48.14	2.63 (2.43–2.83)
Pastoral and farm area	33 482	487	35.52	1.45 (1.32–1.58)
Farm area	17 696	183	13.35	1.03 (0.88–1.18)
Urban area	4108	41	2.99	1.00 (0.70–1.30)

*CI* confidence interval

### Infection in dogs

A total of 7564 faecal samples were collected from dogs in the 74 counties, with 552 positive cases being detected, for a positive rate of 7.30% for the *Echinococcus* coproantigen. There were significant differences across different areas (*χ*^2^ = 44.67, *P* < 0.01), among which the positive rate was highest in Naqu (11.36%) and lowest in Shigatse (4.83%). At the unit of county, the positive rate of *Echinococcus* coproantigen was the highest in Baqing County of Naqu (41.30%). The positive rates of *Echinococcus* coproantigen in different types of areas were statistically significant, of which the positive rate of *Echinococcus* coproantigen in pastoral areas was the highest, at 8.41% (Table 4).

**Table 4:** *Echinococcus* coproantigen ELISA positive rates among dogs in prefectures and by type of area in Tibet Autonomous Region, 2016

	Surveyed population	No. of positive cases	Positive rate/% (95% *CI*)
Prefecture			
Lhasa	1047	66	6.30 (4.83–7.77)
Changdu	1358	78	5.74 (4.50–6.98)
Shannan	1046	107	10.23 (8.39–12.07)
Shigatse	1945	94	4.83 (3.88–5.78)
Naqu	1127	128	11.36 (9.51–13.21)
Ali	423	34	8.04 (5.45–10.63)
Linzhi	618	45	7.28 (5.23–9.33)
Total	7564	552	7.30 (6.71–7.89)
Type of area			
Pastoral area	2224	187	8.41 (7.26–9.56)
Semi-Pastoral and semi-farm area	3033	168	5.54 (4.73–6.35)
Farm area	1637	80	4.89 (3.85–5.93)
Urban area	365	23	6.30 (3.81–8.79)
Total	7564	552	7.30 (6.71–7.89)

*CI* confidence interval

### The prevalence in livestock

A total of 2103 livestock, including yaks, sheep and pigs, from 74 counties in TAR were detected. All surveyed livestock were slaughtered by local residents. The average positive rate of echinococcosis was 11.84%. The highest positive rate of echinococcosis was in Ali Prefecture (28.82%), and the lowest was in Linzhi Prefecture (0.71%). A total of 995 and 1007 yaks and sheep were examined, with positive rates of 9.15 and 15.59%, respectively. The median tooth age of the slaughtered yaks was 7 years old, and the maximum tooth age was 20 years old. The positive rate increased with tooth age (*χ*^2^_trend_ = 57.02, *P* < 0.01). The median tooth age was 5 years old, and the maximum tooth age was 11 years old of 1007 slaughtered sheep. The positive rate increased with tooth age (*χ*^2^_trend_ = 13.99, *P* < 0.01). A total of 101 pigs were examined, and one was positive (Table 5).

**Table 5:** Positive rates of echinococcosis among livestock by clinical examination in Tibet Autonomous Region, 2016

Tooth age by year	Yaks			Sheep			Pigs		
	Surveyed population	No. positive	Positive rate/% (95% *CI*)	Surveyed population	No. positive	Positive rate/% (95% *CI*)	Surveyed population	No. positive	Positive rate/% (95% *CI*)
0–2	118	4	3.39 (0.12–6.66)	50	6	12.00 (2.99–21.01)	34	0	0.00(−)
3–5	222	4	1.80 (0.05–3.55)	562	62	11.03 (8.44–13.62)	64	1	0.16 (0.00–1.14)
6–8	417	41	9.83 (6.97–12.69)	340	76	22.35 (17.92–26.78)	3	0	0.00(−)
9–11	186	25	13.44 (8.54–18.34)	55	13	23.63 (12.40–34.86)	0	0	0.00(−)
12–20	52	17	32.69 (19.94–45.44)	0	0	0.00(−)	0	0	0.00(−)
Total	995	91	9.15 (7.36–10.94)	1007	157	15.59 (13.35–17.83)	101	1	0.99 (0.00–2.92)

-: Not applicable;

*CI*: Confidence interval

### Knowledge of prevention and control

In this survey, a total of 10 799 most boarding students in schools and 7279 local adult-residents in Tibet were enrolled. There were 6036 people who passed the test for knowledge of the prevention and control of echinococcosis, with an awareness rate of 33.39%. Among them, the awareness rates were 38.04% for the students and 26.49% for the local adult residents. There was a significant difference in the awareness rates between these two groups (*χ*^2^ = 1083.40, *P* < 0.01).

## Discussion

Echinococcosis is globally distributed. It is estimated that 91% AE cases of the total number in the world occur in China [12]. Echinococcosis is endemic severely in China, mainly in the western regions, including Inner Mongolia, Tibet, Gansu, Qinghai, Ningxia, and Xinjiang, Sichuan, Yunnan, Shanxi [13–15]. The results of a sampling survey in 2012 showed that the incidence of echinococcosis in the higher-prevalence areas in China, excluding Tibet, was 0.24% [16]. In terms of the geographical environment, the Qinghai-Tibet Plateau in southern Qinghai and western Sichuan, dominated by animal husbandry, is the most endemic area for echinococcosis. The results of B-ultrasonography for the study participants in Qinghai and the Tibetan areas of Sichuan showed prevalence of CE and AE of 3.2 and 3.1%, respectively [17].

This survey used stratified sampling to identify the range and prevalence of echinococcosis in TAR. Our results showed that the prevalence of echinococcosis in people living in the region is 1.66%, which is the highest in China and much higher than the average prevalence of echinococcosis in other parts of China (0.24%). CE was detected in all 74 counties in the region, whereas AE was found in 47% of these counties, accounting for 11.16%. The whole TAR belongs to the Qinghai-Tibet Plateau, which is characterized by a high elevation, a vast territory, and a large number of wild animals. The residents are mainly engaged in pastoral work, and there are many domesticated dogs and livestock on the farms. Stray dogs are ubiquitous, which is an important factor for increased transmission resulting in the high incidence of echinococcosis in this region [18, 19]. At the same time, sanitation facilities in this region, such as those that supply safe water, are lacking. Medical facilities are limited, and qualified medical and health care personnel are few. Living conditions of the residents are generally poor and the residents have a bad health habit. For example, the dogs were fed with raw liver, lung and other organs from slaughtered livestock. Residents know little about the knowledge of echinococcosis prevention and treatment (26.49%). These social factors are also important and challenging issues for the long-term control of echinococcosis in TAR [20–22]. The geographic natural and social conditions in the TAR vary greatly, and the prevalence of echinococcosis was significantly different in different areas. For example, in the areas of Naqu and Ali Prefectures are in the northern Tibet Plateau has with an average elevation of more than 4500 m. These areas represent the major pastoral areas in Tibet, showing prevalence of echinococcosis of 3.37 and 2.31%, respectively, ranking them as the top two areas in the region. In contrast, Shannan City is located in southern Tibet and is a major agricultural area of Tibet, with an average elevation of 3500 m and a lower prevalence of echinococcosis (1.35%).

The survey found that the positive rate among females was significantly higher than that among males (*χ*^2^ = 30.31, *P* < 0.01), which is basically consistent with the results of the survey in Sichuan Tibetan areas and some other study [17, 23–25]. However, there was no significant difference in the positive rate of male and female children under 12 years old in this survey. This may be partly related to the higher exposure of women, in TAR, who are principally responsible for domestic activities, which is the local social custom. Women are in charge of all the major housework, including grazing of animals, feeding of dogs, milking and collecting cow dung, which increases their exposure to contamination by *Echinococcus* eggs in the environment, resulting in a higher risk of echinococcosis.

The results of this survey showed that the positive rates of both males and females increased with age (*χ*^2^_trend_ = 423.95, *P* < 0.01), which is in line with the result of a study on volunteer in the eastern Tibetan Plateau, northwest Sichuan/southeast Qinghai, China, indicating that age and the risk of echinococcosis are related. Echinococcosis, as a chronic infectious disease with a long disease course. Symptoms may develop 5–20 years after infection, and patients may survive for many years after exposure. The elderly may have been exposed to an environment contaminated by *Echinococcus* eggs for an extended period of time, the resulting cumulative risk increases with age, thereby leading to higher prevalence among the elderly. In this survey, in spite of the positive rate increased with age, the highest proportion of case were aged 40–60, mainly related to the larger number and higher positive rate of this age group.

In CE, the active, transitional and inactive cases showed time-related development. Over time, some transitional cases will gradually transform into inactive cases [26]. The cases in this survey in Tibet were sampled on the basis of population. Among the detected CE cases, the active, transitional and inactive cases accounted for 36.2, 13.7 and 50.1%, respectively. This finding is significantly different from the results of a survey in Egypt, in which inactive CE4–5 cases accounted for 17.4% [27]. This may be related to the fact that the cases included in the Egyptian survey were collected from hospitals (the lesions of CE4–5 cases tend to calcify and the cases are less likely to be treated in a hospital) in comparison to this population-based study. TAR had not previously taken any largescale action to prevent, control and treat echinococcosis. The pattern of the progression of the disease is still unclear. However, the results of this study may partly reflect some patterns of CE lesions with very limited prior intervention.

The domestic dog is the most important definitive host for cystic echinococcosis, and raising dog and contact with dogs are key risk factors for human CE [28–31]. In TAR, dogs are common among herdsman families, with 62% of households raising dogs and an average of 1.3 raised dogs per household. There are many stray dogs in both rural and urban areas. Our study found the positive rates of echinococcosis among herders (3.66%) and monks (3.48%) were significantly higher than those of other occupational groups, such as farmers. Monks commonly raise dogs around the temples. The stratified data showed that the positive rate of echinococcosis and the positive rate of *Echinococcus* coproantigen in the pastoral areas were significantly higher than those in the pastoral and farm area, farm area, and urban areas. This indicates a correlation of the positive rates in dogs and the positive rate of echinococcosis in humans.

The present study revealed the characteristics of livestock breeding and different prevalence of slaughtered yaks and sheep in Tibet. Most of the livestock raised in Tibet are yaks and sheep. Many local people believe in Tibetan Buddhism and thus will not kill livestock. Most livestock are mainly used for milking. Large herds area sign of prosperity, with very few livestock being slaughtered or sold; thus, livestock are usually farmed for long periods of time. The survey showed a high average tooth age of slaughtered livestock, with 7 years for yak and 5 years for sheep. This increased the risk of exposure to the echinococcosis eggs and development the risk of echinococcosis in the livestock. In this study, the surveyed livestock were locally slaughtered domestic animals with an average positive rate of 11.84%. The echinococcosis positive rates of yaks and sheep were 9.15 and 15.59%, respectively. As unique livestock animals raised in the Qinghai-Tibet Plateau, yaks have shown a lower echinococcosis prevalence than do sheep in several surveys, in spite of their longer life span, which may be related to the characteristics of the species [32, 33]. This study showed that increasing age of livestock of yaks and sheep was associated with increased risk of echinococcosis. Some other surveys have also shown this association [33–36].

There are still some limitations should be noted. This survey was carried out using portable B-ultrasonography to examine the subjects. Only the abdominal lesions of CE and AE could be detected, whereas lesions in the lungs, brain and other areas outside the abdomen could not be detected; thus, the prevalence detected in the survey among people may be different from the actual case resulting in an artificially lower numerator. The age and gender composition of the surveyed population may be different from the actual situation of local people, because some local people work as migrant workers and might not have been included in the survey. Most of the migrant workers were man between the ages of 30 and 50 years, so the estimation of the prevalence of the population may have selection bias. The survey was done during the cold season in Tibet, with snow accumulation in some areas, it was difficult to investigate the presence of rodents as an intermediate host consequently, data on the prevalence of rodents in this region could not be fully obtained. The livestock were selectively slaughtered, so the positive rate of this survey may be slightly different from the actual prevalence in the livestock.

## Conclusions

Echinococcosis is one of the major public health problems in TAR. It has a high prevalence, a wide range and a mixed typology of the cystic and alveolar types. This seriously threaten the health of the local people and constrains economic development in TAR. Knowledge of the prevention and treatment of echinococcosis among local people is low, and there is a need for better hygienic condition. This combined with a large number of intermediate and terminal hosts all increase the difficulty of the prevention and control of echinococcosis. Rigorous targeted action plans are needed for prevention and control of echinococcosis in Tibet.

## Additional file

Additional file 1: Multilingual abstracts in the five official working languages of the United Nations. (PDF 204 kb)

## Abbreviations

AE: Alveolar echinococcosis
CE: Cystic echinococcosis
DALYs: Disability-adjusted life-years
ELISA: Enzyme-linked immunosorbent assay
TAR: Tibet Autonomous Region
WHO: World Health Organization
