# Supplementary material for: Epidemiological survey of echinococcosis in Tibet Autonomous Region of China
Source: Infect Dis Poverty. 2019 Apr 28;8:29. doi: 10.1186/s40249-019-0537-5 (PMC6487032; doi:10.1186/s40249-019-0537-5)
Supplement: Supplementary file 1 — Multilingual abstracts in the five official working languages of the United Nations. (PDF 204 kb) [file 40249_2019_537_MOESM1_ESM.pdf]

Translation of the abstract into the five official working languages of the United Nations

المسح الوبائي لداء المشوكات في منطقة التبت ذاتية الحكم بالصين

Bin Li, Gongsang Quzhen, Chui-Zhao Xue, Shuai Han, Wei-Qi Chen, Xin-Liu Yan, Zhong-Jie Li, M. Linda Quick, Yong Huang, Ning Xiao, Ying Wang, Li-Ying Wang, Gesang Zuoga, Bianba, Gangzhu, Bing-Cheng Ma, Gasong, Xiao-Gang Wei, Niji, Can-Jun Zheng, Wei-Ping Wu and Xiao-Nong Zhou

نبذة

خلفية: ينتشر داء المشوكات في 10 مقاطعات غرب وشمال الصين. وقد أظهر المسح الوبائي لداء المشوكات في الصين عام 2012 أن معدل الانتشار في أربع مقاطعات بمنطقة التبت ذاتية الحكم البالغة 4.23 بالمائة، يتجاوز بكثير معدل الانتشار في الصين (0.24 بالمائة). لذلك، من المهم فهم مخاطر انتقال هذا المرض لدى الإنسان والحيوان وانتشاره في بمنطقة التبت ذاتية الحكم.

الأساليب: استخدمت طريقة أخذ العينات الطبقية والمتناسبة لاختيار العينات في التبت ذاتية الحكم. وتم فحص السكان المختارين بواسطة التشخيص بالموجات فوق الصوتية، وقد تم اختبار براز الكلاب ل coproantigen مقابل المشوكة الحبيبية باستخدام مقايسة الممتز المناعي المرتبط بالإنزيم. تم فحص الأعضاء الداخلية للحيوانات الأليفة المذبوحة عن طريق الفحص البصري والجس. وقد تم التحقيق في الوعي لمنع ومكافحة داء المشوكات بين السكان والطلاب باستخدام استبيان. أدخلت جميع البيانات باستخدام إدخال مزدوج في قاعدة بيانات Epi Info ، مع تصحيح الخطأ عبر المقارنة المزدوجة الإدخال، وتمت معالجة التحليل الإحصائي لجميع البيانات باستخدام معناها الحزمة الإحصائية للعلوم الاجتماعية 21.0، وتم رسم الخريطة باستخدام ArcGIS 10.1، وتم اختبار البيانات عبراختبار مربع كاي واختبار اتجاه كوكران أرميتاج.

النتائج: تم فحص ما مجموعه 80,384 شخصا، و 7,564 براز كلاب و 2,103 من الأعضاء الداخلية للحيوانات الأليفة المذبوحة. بلغ انتشار داء المشوكات في البشر بالتبت ذاتية الحكم بالمائة1.66٪، وكان المعدل الإيجابي في الإناث (1.92 بالمائة) أعلى بكثير من الذكور (1.41٪ بالمائة) (،χ^2 = 30.31, P < 0.01) ، وكان معدل إيجابي من داء المشوكات إيجابيا المرتبطة بالعمر (χ^2_{trend} = 423.95, P < 0.01) ، والسكان المهنيون الذين لديهم معدلات إيجابية عالية من مرض داء المشوكات كانوا من الرعاة (3.66٪) والرهبان (3.48٪). وصل متوسط المعدل الإيجابي لمركب المشوكة coproantigen في التبت ذاتية الحكم 7.30 ٪. المعدل الإيجابي لداء المشوكات في الماشية للمنطقة بأكملها 11.84٪ بالمائة. بلغ متوسط معدل الوعي بالداء في المنطقة 33.39٪ بالمائة.

الاستنتاجات: يمثل الانتشار العالي لداء المشوكات في جميع أنحاء التبت ذاتية الحكم قلقا خطيرا على صحة الإنسان. وينبغي بذل جهود لوضع خطة عمل للوقاية من داء المشوكة ومكافحته في أقرب وقت ممكن للسيطرة على هذ الداء المستشري والحد من العبء الطبي على السكان

Translated from English version into Arabic by Latifa Haboula, revised by Aalya Al-Beeshi, through

TRANSLATORS
WITHOUT BORDERS

西藏自治区棘球蚴病流行病学调查

Bin Li, Gongsang Quzhen, Chui-Zhao Xue, Shuai Han, Wei-Qi Chen, Xin-Liu Yan, Zhong-Jie Li, M. Linda Quick, Yong Huang, Ning Xiao, Ying Wang, Li-Ying Wang, Gesang Zuoga, Bianba, Gangzhu, Bing-Cheng Ma, Gasong, Xiao-Gang Wei, Niji, Can-Jun Zheng, Wei-Ping Wu et Xiao-

Nong Zhou

引言：棘球蚴病在中国西部和北部 10 个省（自治区、建设兵团）流行。2012 年中国棘球蚴病流调情况显示，西藏自治区调查的 4 个县人群平均患病率为 4.23%，远高于中国（0.24%）的平均患病率。因此，了解西藏棘球蚴病在人和动物中的传播风险和流行情况是很重要的。
方法：采用分层整群抽样法对人群进行进行抽样。对选定的居民进行 B 超检查，并用酶联免疫吸附试验检测犬粪棘球绦虫抗原。采用目视和触诊法对屠宰的家畜内脏进行检查。采用问卷调查的方法，对居民和学生进行棘球蚴病防治知识和行为调查。Epi Info 数据库双录入法进行数据录入和质量控制，使用 SPSS 21.0 对数据进行统计分析，利用 ARCGIS 10.1 绘制地图，采用卡方检验和趋势检验对数据进行检验。
结果：本调查共计检查 80384 人、7564 份犬粪、2103 副家畜内脏。西藏自治区人群棘球蚴病的患病率为 1.66%，其中女性（1.92%）明显高于男性（1.41%），（χ^2 = 30.31，P < 0.01），人群棘球蚴病检出率与年龄呈正相关（$\chi^2_{\text{趋势}}$=423.95，P < 0.01），不同职业人群中牧民（3.66%）和僧侣（3.48%）的棘球蚴病检出率较高。西藏自治区犬粪抗原平均阳性率为 7.30%，家畜棘球蚴病阳性率为 11.84%，棘球蚴病防治知识和行为调查平均知晓率为 33.39%。
结论：西藏棘球蚴病流行严重。尽快制定防治棘球蚴病行动计划，控制地方性棘球蚴病流行，减轻人民群众的医疗负担。

Translated from English version into Chinese by Chui-Zhao Xue

Étude épidémiologique de l’échinococcose dans la Région autonome du Tibet, en Chine

Bin Li, Gongsang Quzhen, Chui-Zhao Xue, Shuai Han, Wei-Qi Chen, Xin-Liu Yan, Zhong-Jie Li, M. Linda Quick, Yong Huang, Ning Xiao, Ying Wang, Li-Ying Wang, Gesang Zuoga, Bianba, Gangzhu, Bing-Cheng Ma, Gasong, Xiao-Gang Wei, Niji, Can-Jun Zheng, Wei-Ping Wu et Xiao-Nong Zhou

Résumé
Contexte : l’échinococcose est présente dans 10 provinces de l’ouest et du nord de la Chine. Une étude épidémiologique menée en Chine en 2012 a montré que sa prévalence moyenne dans quatre circonscriptions de la Région autonome du Tibet était de 4,23 %, très au-dessus de la prévalence moyenne en Chine (0,24 %). Il est important de comprendre les risques de transmission et la prévalence de l’échinococcose parmi les populations humaines et animales de la Région autonome du Tibet.
Méthodes : nous avons utilisé une méthode d’échantillonnage stratifiée et proportionnelle pour sélectionner des échantillons provenant de la Région autonome du Tibet. Les habitants sélectionnés ont été examinés par échographie en mode B et des déjections canines ont été analysées à la recherche du coproantigène canin dirigé contre l’*Echinococcus spp.* par immunoadsorption enzymatique (ELISA). Les organes internes d’animaux domestiques abattus ont été examinés visuellement et par palpation. La sensibilisation à la prévention et à la lutte contre l’échinococcose parmi les habitants et en milieu scolaire a été évaluée à l’aide d’un questionnaire. Toutes les données ont été saisies à l’aide d’une double entrée dans la base de données Epi Info, avec correction des erreurs par comparaison des doubles entrées. L’analyse statistique de toutes les données a été

réalisée avec SPSS 21.0 et la carte a été tracée avec ArcGIS 10.1. Les données ont été vérifiées avec le test du chi carré et le test de tendance Cochran-Armitage.

Résultats : au total, 80 384 personnes, 7564 déjections canines et 2103 organes internes d’animaux domestiques abattus ont été examinés. La prévalence de l’échinococcose parmi la population humaine de la Région autonome du Tibet était de 1,66 %, avec un taux de résultats positifs significativement plus élevé chez les femmes (1,92 %) que chez les hommes (1,41 %), (χ^2 = 30,31, P < 0,01). Le taux de cas positifs était associé positivement à l’âge ($\chi^2_{tendance}$ = 423,95, P < 0,01) et les métiers présentant des taux élevés d’échinococcose étaient les éleveurs (3,66 %) et les moines (3,48 %). Le taux moyen d’échantillons positifs au coproantigène d’*Echinococcus* était de 7,30 % dans la Région autonome du Tibet. Le taux de cas positifs d’échinococcose parmi le bétail de toute la région était de 11,84 %. Le taux moyen de sensibilisation de la population de la région à l’échinococcose était de 33,39 %.

Conclusions : la prévalence de l’échinococcose est élevée dans toute la Région autonome du Tibet, où elle représente un important problème de santé publique. Des efforts sont nécessaires pour élaborer un plan de prévention et de lutte contre cette parasitose dans les plus brefs délais, afin de juguler l’épidémie et de réduire son fardeau médical pour la population.

Translated from English version into French by Suzanne Assenat, Revised by Eric Ragu, through

TRANSLATORS
WITHOUT BORDERS

Эпидемиологическое исследование очагов эхинококкоза в Тибетском автономном районе Китая

Бинь Ли, Гунсан Цюйчжень, Чуи-Чжао Сюэ, Шуай Хань, Вэй-Ци Чэнь, Синь-Лю Янь, Чжун-Цзе Ли, М. Линда Квик, Юн Хуан, Нин Сяо, Ин Ван, Ли-Ин Ван, Гэсан Цзога, Бианьба, Ганчжу, Бин-Чэн Ма, Гасун, Сяо-Ган Вэй, Ницзи, Цань-Цзюнь Чжэн, Вэй-Пин У, Сяо-Нун Чжоу

Аннотация

Справочная информация: Эхинококкоз широко распространен в 10 провинциях Западного и Северного Китая. По результатам эпидемиологического исследования очагов эхинококкоза в Китае, проведенного в 2012 г., средний показатель распространенности заболевания в четырех округах Тибетского автономного района (ТАР) составляет 4,23%, что значительно превышает средний показатель распространенности заболевания по Китаю в целом (0,24%). Таким образом, необходимо выяснение возможных способов заражения и уровня заболеваемости эхинококкозом среди людей и животных в ТАР.

Методы: Для отбора проб в ТАР был использован метод районированной и пропорциональной выборки. Обследование населения проводилось с применением эхографии в В-режиме. Наличие копроантигенов *Echinococcus* spp. в фекалиях собак выявлялось с помощью фермент-связанного иммуносорбентного исследования. Кроме того,

проводилось визуальное и пальпационное исследование внутренних органов забитого домашнего скота. Выяснение уровня осведомленности о профилактике эхинококкоза и методах борьбы с ним среди местного населения и студентов проводилось методом опроса. Полученные результаты были введены методом двойного ввода данных в базу Epi Info с последующей коррекцией ошибок, выявленных при сравнении записей. Для статистического анализа данных использовалась компьютерная программа SPSS 21.0. При составлении карты применялась компьютерная программа ArcGIS 10.1. Тестирование данных проводилось посредством проверки на соответствие по критерию хи-квадрат и теста тренда Кохрана-Армитажа.

Результаты: В общей сложности было осмотрено 80 384 человек, исследовано 7 564 образцов фекалий и 2 103 образцов внутренних органов забитого домашнего скота. Выявленный уровень распространения эхинококкоза среди населения ТАР составляет 1,66 %, причем коэффициент позитивности среди женщин (1,92 %) значительно выше, чем среди мужчин (1.41%), ($\chi^2 = 30.31$, $P < 0.01$). Коэффициент позитивности при эхинококкозе положительно коррелирует с возрастом ($\chi^2_{trend} = 423.95$, $P < 0.01$). Профессиональными группами с наиболее высоким коэффициентом позитивности при эхинококкозе являются скотоводы и монахи, среди которых данный коэффициент составляет 3.66 % и 3.48 % соответственно. Средний коэффициент позитивности для копроантигенов *Echinococcus* в ТАР составляет 7,30 %. Коэффициент позитивности при эхинококкозе у домашнего скота по исследуемому району в целом составляет 11.84 %. Средний уровень осведомленности об эхинококкозе составляет по региону 33.39 %.

Выводы: Широкая распространенность эхинококкоза в ТАР, выявленная в ходе исследования, представляет серьезную угрозу здоровью населения. Для профилактики эхинококкоза и борьбы с ним необходимо скорейшее составление плана действий с целью борьбы с эпидемией эхинококкоза и уменьшения финансового бремени, связанного с расходами на лечение, среди населения.

Translated from English version into Russian by Anastasiia Dobrosynets, revised by Tatiana Fridman, through

TRANSLATORS WITHOUT BORDERS

Estudio epidemiológico de la equinococosis en la Región Autónoma del Tíbet, en China

Bin Li, Gongsang Quzhen, Chui-Zhao Xue, Shuai Han, Wei-Qi Chen, Xin-Liu Yan, Zhong-Jie Li, M. Linda Quick, Yong Huang, Ning Xiao, Ying Wang, Li-Ying Wang, Gesang Zuoga, Bianba, Gangzhu, Bing-Cheng Ma, Gasong, Xiao-Gang Wei, Niji, Can-Jun Zheng, Wei-Ping Wu y Xiao-Nong Zhou

Resumen

Antecedentes: la equinococosis prevalece en 10 provincias del oeste y del norte de China. En el

año 2012, un estudio epidemiológico de la equinococosis en China demostró que la prevalencia media de la enfermedad en cuatro condados de la Región Autónoma del Tíbet (RAT) era de un 4,23 %, un valor mucho más elevado que el de la prevalencia media en China (0,24 %). Es importante comprender los riesgos de transmisión y la prevalencia de la equinococosis en la RAT, tanto en humanos como en animales.

Métodos: para la selección de muestras en la RAT se empleó un método de muestreo estratificado y proporcionado. Se examinó a los residentes seleccionados con un diagnóstico ultrasonográfico en modo B, al mismo tiempo que se utilizó un ensayo de inmunoabsorción enzimática (ELISA) en las heces de perros para la detección del coproantígeno canino contra *Echinococcus* spp. Los órganos internos de los animales domésticos sacrificados se analizaron mediante examen visual y palpación. Se utilizó un cuestionario para investigar el conocimiento de la prevención y control de la equinococosis entre los residentes y los estudiantes. Los datos se introdujeron con doble entrada en la base de datos Epi info, con corrección de errores mediante la comparación de la doble entrada. Se utilizó SPSS 21.0 para el análisis estadístico de todos los datos, el mapa se cartografió con ArcGIS 10.1 y los datos se evaluaron mediante el test de chi-cuadrado y el test de tendencia de Cochran-Armitage.

Resultados: se analizó un total de 80 384 personas, 7 564 heces de perro y 2 103 órganos internos de animales domésticos sacrificados. La prevalencia de la equinococosis en seres humanos en la RAT fue de 1,66 %; el índice de positivo en mujeres (1,92 %) fue significativamente mayor que el de los hombres (1,41 %), ($\chi^2 = 30,31$, $P < 0,01$); el índice de positivo de equinococosis estuvo positivamente asociado con la edad (*tendencia* $\chi^2 = 423,95$, $P < 0,01$) y las poblaciones ocupacionales con mayores índices positivos de equinococosis fueron las de pastores (3,66 %) y los monjes (3,48 %). El índice positivo medio del coproantígeno *Equinococcus* en la RAT fue del 7,30 %. El índice positivo de equinococosis en el ganado para toda la región fue del 11,84 %. La tasa media de conocimiento de la equinococosis en la región fue del 33,39 %.

Conclusiones: se ha encontrado una alta prevalencia de la equinococosis en la RAT, lo que supone una grave preocupación para la salud humana. Se deben realizar esfuerzos para desarrollar lo antes posible un plan de acción para la prevención y el control de la equinococosis, con el fin de controlar la endemia de la equinococosis y reducir la carga de los gastos médicos en la población.

Translated from English version into Spanish by Celia Martinez, Revised by Mayra León, through

TRANSLATORS
WITHOUT BORDERS
